# Evaluation of a Novel Lateral Emitting Laser Fiber for Near-Infrared Photoimmunotherapy

**DOI:** 10.3390/cancers16142558

**Published:** 2024-07-17

**Authors:** Motofumi Suzuki, Hisataka Kobayashi, Hirofumi Hanaoka

**Affiliations:** 1 Division of Fundamental Technology Development, Near InfraRed Photo-ImmunoTherapy Research Institute, Kansai Medical University, Hirakata 573-1010, Osaka, Japan; suzukimo@hirakata.kmu.ac.jp; 2 Molecular Imaging Branch, Center for Cancer Research, National Cancer Institute, National Institutes of Health, Bethesda, MD 20892-1088, USA; kobayash@mail.nih.gov

**Keywords:** cancer treatment, laser fiber, near-infrared photoimmunotherapy

## Abstract

**Simple Summary:**

Near-infrared photoimmunotherapy (NIR-PIT) is a recently developed cancer therapy that uses NIR light and conjugates of a tumor-targeting monoclonal antibody and phthalocyanine dye. In clinical practice, frontal and cylindrical diffusers are used for NIR illumination. However, the light illumination of a narrow space is technically difficult with such diffusers. Therefore, we evaluated the usefulness of a lateral illumination system using lateral emitting laser (LEL) fiber. The LEL fiber was capable of illuminating only a certain area in a lateral direction. NIR-PIT with an LEL fiber showed cytotoxic effects in vitro and suppressed tumor proliferation in vivo. Moreover, the LEL fiber delivered NIR light to an entire tumor more uniformly than a frontal diffuser in a narrow-space model. These results suggest that the LEL fiber can be used for NIR-PIT and is suitable for NIR light illumination in a narrow space.

**Abstract:**

Near-infrared photoimmunotherapy (NIR-PIT) is a new cancer therapy that uses NIR light and conjugates of a tumor-targeting monoclonal antibody and phthalocyanine dye. In clinical practice, frontal and cylindrical diffusers are the only options for NIR illumination. However, illumination in a narrow space is technically difficult with such diffusers. Therefore, we evaluated a lateral illumination system using a lateral emitting laser (LEL) fiber. The LEL fiber illuminated a certain area in a lateral direction. NIR-PIT with an LEL fiber reduced luciferase activity in a light-dose-dependent manner in A431-GFP-luc cells in vitro and significantly suppressed tumor proliferation in a xenograft mouse model. To evaluate the usefulness of the LEL fiber in the illumination of a narrow space, a tumor was illuminated from the inside of a cylinder, mimicking a narrow space, and the fluorescence intensity in the tumor was monitored. In the frontal diffuser, NIR light was unevenly delivered and little light reached a distal tumor area from the illuminated side. By contrast, the LEL fiber allowed a uniform illumination of the entire tumor, and a loss of fluorescence was observed even in distal areas. These findings suggested that the LEL fiber can be used for NIR-PIT and is suitable for NIR light illumination in a narrow space.

## 1. Introduction

Near-infrared photoimmunotherapy (NIR-PIT) is a recently developed cancer therapy that uses NIR light at 690 nm and conjugates of a tumor-targeting monoclonal antibody and photo-absorbing phthalocyanine dye (IRDye700DX; IR700) [1]. IR700 is chemically decomposed by NIR light irradiation, which leads to aggregation of conjugates. When this change occurs on the membrane of target cells, it causes lethal membrane damage and leads to immunogenic cell death. Importantly, such conjugates and NIR light are minimally toxic; cell death is induced only when this conjugate binds to cancer cell antigens and is exposed to NIR light [2]. Therefore, NIR-PIT has the potential to provide a minimally invasive but highly selective treatment for many types of cancer. A global phase III randomized controlled trial of NIR-PIT was started in 2018 and is currently ongoing (NCT03769506). Moreover, NIR-PIT targeting the epidermal growth factor receptor (EGFR) using a cetuximab-IR700 conjugate was approved in Japan in September 2020 for the treatment of recurrent head and neck squamous cell carcinoma. In addition, NIR-PIT can target any surface membrane protein, regardless of carcinoma type. Therefore, preclinical experiments have shown that NIR-PIT can treat various types of cancers such as lung, pancreatic, ovarian, prostate, breast, gastric, colorectal, and bladder cancer, by targeting not only EGFR but also various molecules like human epidermal growth factor receptor 2, carcinoembryonic antigen, and prostate-specific membrane antigen [2]. Furthermore, the usefulness of NIR-PIT in targeting other than cancer cells, including cancer-associated fibroblasts and regulatory T cells, has been reported.

In NIR-PIT, since NIR light exposure is an essential part of the treatment, the use of an appropriate illumination device is important to maximize therapeutic efficacy. Light at a wavelength of approximately 700 nm penetrates approximately 1 cm into tissue [3]; thus, superficial tumors, such as those of the skin and mouth, can be easily treated by surface illumination with a frontal diffuser. For a more deeply located tumor, needle catheters are placed into the tumor and light is applied from inside the tumor with a cylindrical light diffuser [4,5]. Moreover, the use of an illuminating cylindrical light diffuser through an endoscope is effective in a preclinical model of gastrointestinal tract tumors and head and neck cancers [6,7]. Endoscopic illumination can be applied to various types of cancers, such as esophageal, colorectal, and urinary tumors [8,9]. Currently, forward-aiming laser fibers are used in endoscopic illumination in photodynamic therapy that uses laser illumination similarly to NIR-PIT [10,11]. Therefore, it is expected that the frontal diffuser currently used in clinical practice will be employed for illumination in NIR-PIT.

Although laser irradiation through an endoscope is a promising method, several challenges remain: A cylindrical light diffuser can irradiate in a 360-degree direction but it is difficult to limit the irradiation area. In addition, the NIR light is irradiated into normal areas where it is not required. Also, since EGFR is expressed, to some extent, in normal mucosal cells, excess irradiation to normal areas might cause side effects [12,13,14]. In the case of a frontal diffuser, when treating a prominent tumor, the laser energy is delivered only at the proximal side. Moreover, uniform illumination from an oblique angle is difficult in organs with narrow lumens, such as the esophagus [15]. Thus, a new type of diffuser is required to irradiate various areas, and a lateral emitting laser (LEL) fiber has potential in this regard. In the present study, we evaluated the usefulness of a novel LEL fiber for NIR-PIT by comparing forward-aiming laser fibers in vitro and in vivo.

## 2. Materials and Methods

### 2.1. Reagents and Antibodies

A water-soluble, silicon-phthalocyanine derivative IRDye700DX N-hydroxysuccinimide ester (IR700-NHS) was purchased from LI-COR Bioscience (Lincoln, NE, USA). Cetuximab, a chimeric monoclonal antibody directed against EGFR, was obtained from Merck Biopharma (Tokyo, Japan). All other chemicals were of reagent grade.

### 2.2. Beam Profile Determination

An LEL fiber was kindly supplied by Furukawa Electric Co., Ltd. (Tokyo, Japan). A microlens at the tip of the fiber allows lateral illumination. The beam profile of the laser with the LEL fiber was measured with a working distance of 4.5 mm using a BGP-USB-SP928-OSI beam profiling camera (Ophir; Tokyo, Japan). The laser was driven using an LDX-3230-685-HHL-400 semiconductor laser diode (LDX Optronics Inc; Maryville, TN, USA). The data were acquired in two separate sessions and a heatmap was drawn since the laser illumination field exceeded the active area of the beam profiler. Light intensity is shown as a relative value with background as zero.

### 2.3. Synthesis of IR700-Conjugated Cetuximab

Cetuximab (5 mg, 34.2 nmol) was incubated with IR700 NHS ester (669.2 μg, 342.5 nmol) in 0.1 M Na_2_HPO_4_ (pH 8.5) for 24 h at room temperature. The mixture was purified in a Sephadex G25 column (PD-10; GE Healthcare, Piscataway, NJ, USA) to obtain IR700-conjugated cetuximab (Cet-IR700). The protein concentration was determined with a protein assay BCA reagent (Fujifilm Wako Pure Chemical Industries; Osaka, Japan) by measuring the absorption at 562 nm with a spectrophotometer (V-730Bio; JASCO, Tokyo, Japan). The concentration of IR700 was measured by a spectrophotometer at an absorption wavelength of 689 nm to determine the number of fluorophore molecules conjugated to cetuximab. The synthesis was controlled so that an average of three IR700 molecules were bound to a single antibody.

### 2.4. Cell Culture

Human epidermoid carcinoma A431 cell lines expressing green fluorescence protein (GFP) and firefly luciferase (A431-GFP-luc) were established by Dr. Kobayashi’s laboratory (National Institutes of Health, Bethesda, MD, USA). The cells were maintained in Dulbecco’s modified Eagle’s medium (DMEM; Fujifilm Wako Pure Chemical Industries) supplemented with 10% (*v*/*v*) fetal bovine serum (FBS; GE Healthcare, South Logan, UT, USA) and antibiotics (100 μg/mL penicillin and streptomycin) at 37 °C in a humidified atmosphere of 5% CO_2_.

### 2.5. In Vitro NIR-PIT

Since the thickness of a normal culture dish was more than 10 mm, cells were seeded onto the inner lid of a culture dish coated with collagen (Cellmatrix Type I-C; Nitta Gelatin; Osaka, Japan) and then cultured overnight. After incubation with Cet-IR700 (10 μg/mL) for 1 h at 37 °C, NIR light was irradiated onto the cells using an LEL fiber from a height of 10 mm with an ML7710 laser system (Modulight; Tampere, Finland) at a dose of up to 32 J/cm^2^. The actual power density was measured with an optical power meter (PM 100; Thorlabs, Newton, NJ, USA). For bioluminescence imaging (BLI), a medium containing D-luciferin (150 μg/mL) was administered to cells 1 h after NIR-PIT. Subsequently, BLI was performed using an IVIS Spectrum In Vivo Imaging System (PerkinElmer, Waltham, MA, USA).

### 2.6. In Vivo NIR-PIT

All animal experiments were performed according to the established guidelines of the “Law for The Care and Welfare of Animals in Japan” and approved by the Ethics Committee on Animal Experiments at Kansai Medical University (22-001 and 23-022). All animal experiments were conducted with a minimum of five mice. A431-GFP-luc cells were prepared by suspending 5 × 10^6^ cells in 100 μL phosphate-buffered saline; the cell suspension was injected subcutaneously into 6-week-old female BALB/c nu/nu mice (The Jackson Laboratory Japan, Inc., Yokohama, Japan). When the tumor volume exceeded approximately 100 mm^3^, the mice were randomized into three groups: (i) no treatment; (ii) NIR light (100 J/cm^2^) without Cet-IR700; and (iii) NIR light (100 J/cm^2^) with Cet-IR700 (NIR-PIT treatment). The NIR light exposure was performed using an LEL fiber with an ML7710 laser system. Imaging for IR700 fluorescence before and after NIR light exposure was conducted with a Pearl Trilogy small animal imaging system (LI-COR Biosciences) 24 h after the injection of Cet-IR700. Tumor volume was measured using calipers and calculated using the following formula: size (mm^3^) = length (mm) × width (mm) × width (mm) × 0.5.

### 2.7. Evaluation of NIR Light Penetration into Tumors

To mimic the NIR light illumination of tumors in narrow lumens, a narrow acrylic cylinder (Φ 20 mm) with a square hole was prepared [16]. Mice were positioned so that each tumor was in the hole. Laser fibers were inserted in the cylinder and NIR light was illuminated at 100 J/cm^2^. Comparisons were made for the following three different irradiation methods: LEL fiber illumination, frontal illumination with a frontal diffuser (Medlight; Ecublens, Switzerland), and angle illumination with a frontal diffuser bent 30 degrees to the cylinder, more closely resembling clinical conditions. For lateral illumination, the LEL fiber was placed directly above the tumor and irradiated from a height of 1 cm. For angle illumination, the light was illuminated using a frontal diffuser with the angle fixed at 30 degrees to deliver the light on the entire tumor. Five mice were used in each group.

Using a side view, IR700 fluorescence imaging was conducted with a LuminousQuester NI Near-Infrared Light Imaging System (Shimadzu Corporation, Kyoto, Japan). The camera of the system was placed approximately 50 cm from a tumor-bearing mouse [17]. For the comparison of pre- and post-treatment fluorescence imaging, fluorescence images of tumors were obtained before and after therapeutic NIR light illumination. To avoid the therapeutic effects of NIR light illumination for fluorescence imaging, illumination was performed for only 5 s from the same side as the imaging camera using a laser emitting light from a 690 nm continuous wave laser (MLL-III-690, CNI Technology, Changchun, China) with output timing controllable by a TTL control unit (Shimadzu Corporation). Three regions of interest (ROI) for each tumor area were set, and the pixel values were measured using ImageJ Fiji software (Version: 2.14.0/1.54f) [18]. The fluorescence intensity of pre-treatment was set to 100% and the loss of fluorescence was calculated.

### 2.8. Statistical Analysis

All results are expressed as mean ± standard deviation (SD) of the mean. Statistical analysis was carried out using GraphPad Prism 9.0 (GraphPad Software, Inc., San Diego, CA, USA). Comparisons of the two groups were performed using Student’s *t*-tests. For multiple comparisons, a Tukey–Kramer test was used. The minimum level of significance was set at *p* < 0.05.

## 3. Results

### 3.1. Properties of LEL Fiber

**Figure 1:**
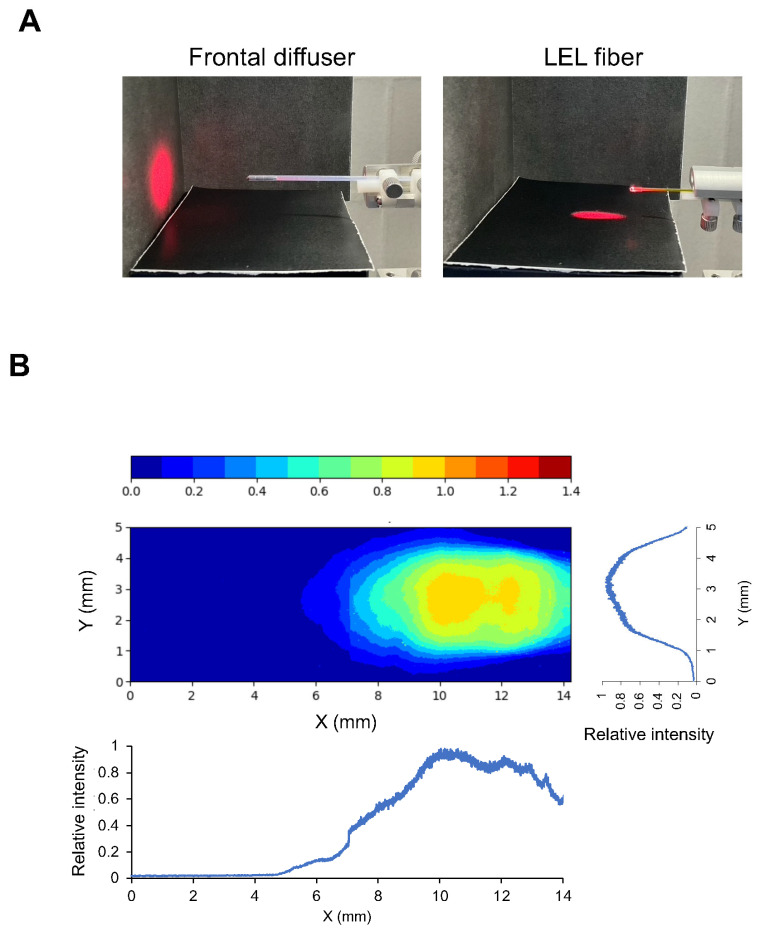
Properties of an LEL fiber. (**A**) Photographs of a laser spot using a frontal diffuser and LEL fiber. NIR light illumination with an LEL fiber was performed at a 10 mm height. (**B**) Uniformity of the illumination field provided by the LEL fiber. A heatmap image of the illumination intensity distribution viewed from above and a graph of the relative intensity profiles in the line of the fiber and perpendicular to the line of the fiber at a central illumination area, respectively.

As shown in Figure 1A, the frontal diffuser is illuminated in the forward direction only. By contrast, LEL fibers enabled illumination in a lateral direction only. The NIR light intensity using the LEL fiber on the illuminated field was measured with a working distance of 4.5 mm (Figure 1B). This beam profile showed that the LEL fiber enables uniform illumination in only a certain area to some extent.

### 3.2. In Vitro NIR-PIT Assessed by BLI

**Figure 2:**
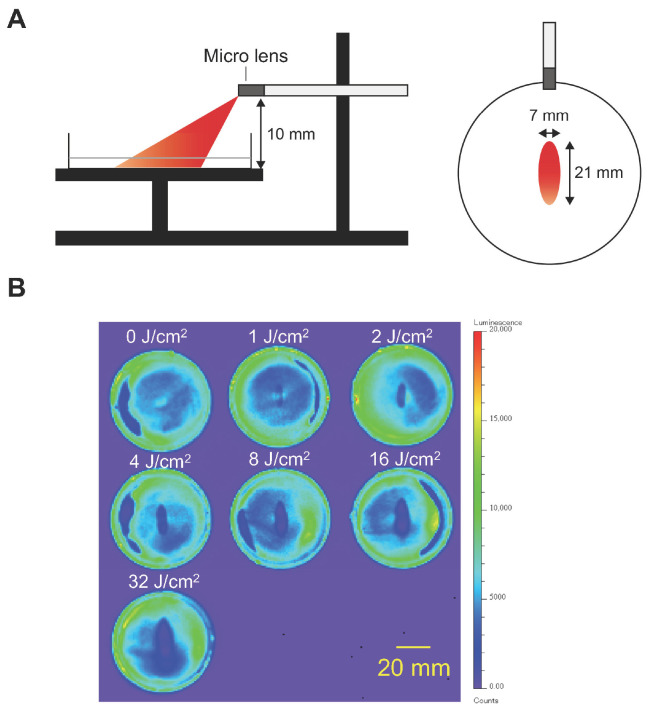
In vitro NIR-PIT in A431-GFP-luc. NIR light was illuminated using an LEL fiber in A431-GFP-luc cells treated with Cet-IR700. The cytotoxicity of NIR-PIT was assessed by bioluminescence imaging. (**A**) Schematic of in vitro NIR-PIT experimental settings. The illumination area from a 10 mm height was an ellipse, with a length of 21 mm and width of 7 mm. (**B**) Bioluminescence images of each dish after NIR-PIT at various doses in A431-GFP-luc cells. Scale bar, 20 mm.

The cytotoxicity of NIR-PIT with an LEL fiber was assessed in Cet-IR700-treated cancer cells. Assuming illumination in a narrow space, such as the esophagus, the illumination was performed at a height of 10 mm. As shown in Figure 2A, the illumination area was ellipse-shaped, with a length of 21 mm and a width of 7 mm. After illumination, luciferase activity in A431-GFP-luc cells decreased within a certain area (Figure 2B). These results suggested that laser illumination with an LEL fiber induced a photochemical reaction and exhibited a cytotoxic effect on A431-GFP-luc cells. The area of the treatment effect was expanded in a dose-dependent manner up to 8 J/cm^2^ (R^2^ = 0.9877). However, above 16 J/cm^2^, the effective area was not expanded beyond a certain size, which is consistent with the measured beam profile.

### 3.3. Therapeutic Efficacy of NIR-PIT In Vivo

**Figure 3:**
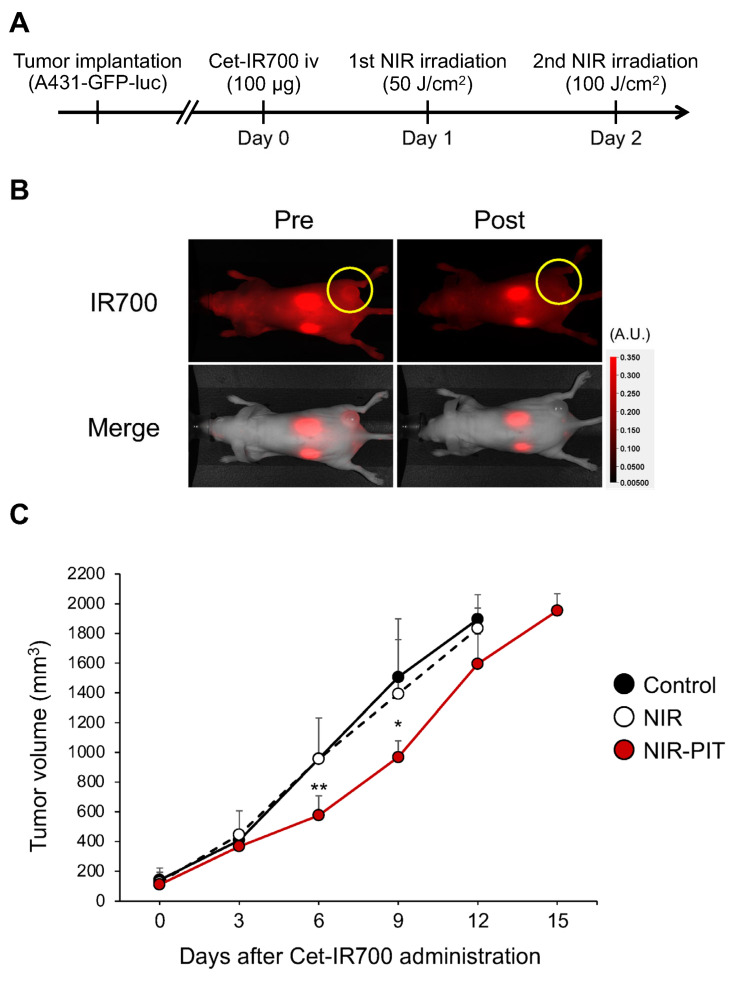
In vivo NIR-PIT using an LEL fiber in an A431-GFP-luc tumor-bearing mouse model. (**A**) Schematic diagram of Cet-IR700 injection and NIR light exposure. (**B**) Fluorescence images on Day 1 before and immediately after NIR-PIT. Yellow circles indicate tumor beds. (**C**) Tumor growth inhibition by NIR-PIT with LEL fiber. The graph represents tumor volumes after treatment. Data are expressed as the mean ± standard deviation (SD). * *p* < 0.05, ** *p* < 0.01 versus control (Tukey–Kramer test).

The therapeutic effect of lateral illumination with an LEL fiber was examined in a tumor-bearing mouse model. The treatment schema is shown in Figure 3A. The accumulation of Cet-IR700 in an A431-GFP-luc tumor was observed 24 h after intravenous injection (Figure 3B). In the NIR-PIT group, the IR700 fluorescence signal disappeared after NIR light exposure due to structural changes of the IR700 dye [19]. These results suggested that NIR light with an LEL fiber can induce NIR-PIT in vivo. After treatment, tumor growth was significantly suppressed in the NIR-PIT group by Days 6 and 9 (Figure 3C). No change in tumor volume was observed in the NIR-irradiated group only. These results suggested that the LEL fiber enabled the therapeutic efficacy of NIR-PIT in vivo as well as the use of a frontal light distributor used in previous experiments [20].

### 3.4. Comparison of Forward- and Lateral-Aiming Illumination

**Figure 4:**
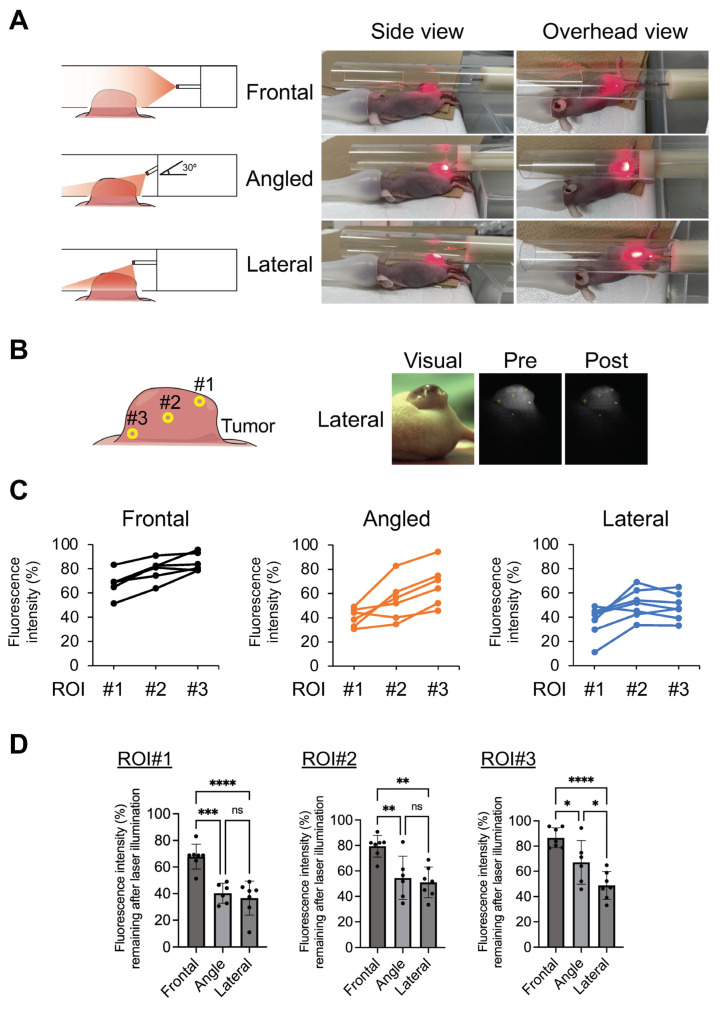
Evaluation of light penetration in a tumor model. Penetration of NIR light was compared using a frontal diffuser and LEL fiber. A fiber was fixed in the center of a 20 mm diameter cylinder, which mimicked a narrow lumen, with a hole to fit the tumor. The tumor was then illuminated by NIR light. In angled illumination, the frontal diffuser was fixed to the tube at an angle of 30 degrees. (**A**) Schematic and visual images of NIR illumination. (**B**) Schematic of three regions of interest (#1: shallow and proximal areas, #2: middle areas, and #3: deep and distal areas). Representative visual and fluorescence images of a tumor in the NIR-PIT with LEL fiber mouse treatment group. (**C**) Graphs represent the individual fluorescence intensity remaining after the illumination of each mouse at ROI #1–3. The fluorescence intensity of pre-treatment was set to 100% and the loss of fluorescence was calculated. (**D**) Graph represents fluorescence intensity remaining after illumination. Data are expressed as the mean ± SD. * *p* < 0.05, ** *p* < 0.01, *** *p* < 0.001, **** *p* < 0.0001 versus control (Tukey–Kramer test).

To undertake evaluations in a more clinically relevant study, an illumination model in a narrow cylinder was prepared for an in vivo study. A fiber was fixed in the center of the cylinder and the NIR illumination of a prominent tumor was undertaken (Figure 4A). In these experiments, the penetration of NIR light into the tumor using a lateral emitting fiber was compared with that of an existing frontal emitting diffuser. Moreover, oblique angle-aiming illumination was undertaken using a frontal emitting fiber, assuming endoscopic illumination using a frontal diffuser in clinical practice. Although NIR illumination was undertaken at a distance from the tumor to illuminate the entire tumor with a forward-aiming diffuser, it was obvious that the light was barely delivered to the distal regions of the tumor (Figure 4A). In angled illumination, increased delivery of the NIR light occurred compared to that in frontal illumination; however, the illumination was uneven and more was delivered to the proximal region. On the other hand, the NIR light was delivered to the entire tumor when using lateral illumination. To perform a more quantitative analysis, the fluorescence intensity of IR700 was compared before and after therapeutic NIR illumination (Figure 4B–D). After obtaining images, three ROIs (#1: shallow and proximal area, #2: middle area, and #3: deep and distal area) were set on the tumor and the loss of IR700 fluorescence by NIR-PIT was compared. In shallow (ROI #1) and intermediate (ROI #2) regions, the loss of fluorescence was significantly induced by angled and lateral illumination compared with frontal illumination (#1: frontal, 67.8 ± 9.3%; angled, 40.3 ± 7.5%; lateral, 36.6 ± 12.8%; and #2: frontal, 79.5 ± 8.5%; angled, 54.5 ± 17.1; lateral, 51.1 ± 12.1%). However, no significant differences were found between angle and lateral illuminations. In contrast, in deep (ROI #3) regions, lateral illumination occurred with more of a loss of fluorescence compared with angled illumination (#3: frontal, 86.5 ± 7.9%; angled, 67.0 ± 17.4%; lateral, 48.8 ± 10.9%). In a comparison within each tumor, the loss of fluorescence between ROIs #2 and #3 was almost the same as for the lateral illumination group. A decrease in the loss of fluorescence between ROI #2 and ROI #3 was found for the angled illumination group. These results suggested that NIR illumination with an LEL fiber allowed the uniform illumination of the entire tumor and is suitable for NIR light illumination in a narrow space compared to a frontal emitting laser.

## 4. Discussion

As a therapy, NIR-PIT stands out as a promising cancer treatment strategy, with numerous drugs developed for photoimmunotherapy [2]. Despite this progress, the development of new diffusers for NIR light exposure, a crucial aspect of the treatment, has lagged. The LEL fiber assessed in this study illuminated a specific lateral area, inducing photochemical reactions and demonstrating cytotoxic effects on tumor cells. In vivo tumor-bearing models further demonstrated the therapeutic efficacy of NIR-PIT with the LEL fiber. The cytotoxic effects of NIR-PIT do not vary by illumination method as long as the same amount of light is irradiated. Therefore, the LEL fiber would enable the induction of therapeutic efficacy as same as the diffuser used in clinical practice. This indicates that the LEL fiber possesses sufficient properties that are effective for NIR-PIT use.

Gastrointestinal endoscopy has been widely used for diagnosis and treatment in clinical practice [21,22]. Therefore, illumination with endoscopic NIR light illumination makes it possible to expand the cancer indications of NIR-PIT [9]. However, endoscopic illumination using a forward-aiming diffuser is hard to apply to some tumors in narrow spaces. In the current study of a cylinder model, the forward-aiming illumination of NIR light required a longer focal length to illuminate the entire tumor. It is difficult to deliver adequate NIR light for treatment. Although we succeeded in shortening the focal length after illumination at an angle of 30 degrees, uniform light illumination was difficult to achieve, and most of the NIR light was delivered to a proximal area. Furthermore, although our cylinder model was 2 cm in diameter, actual anatomical lumens are often much thinner, and a 30-degree bend is assumed to be difficult. By contrast, NIR light illumination with an LEL fiber illuminated the entire tumor, showing that light could be delivered to distal depths. Even if the position of the fiber was closer to the tumor in a narrower lumen, it would be possible to irradiate the tumor without issue because of the lateral illumination. Accordingly, an LEL fiber is suitable for NIR light illumination in a narrow space and may maximize the therapeutic efficacy of NIR-PIT.

Head and neck cancers are relatively easy to illuminate with NIR light using existing light diffusers. A frontal diffuser is used for surface illumination, and a cylindrical diffuser is used for deep lesions, sometimes with the assistance of an imaging modality such as endoscopes, ultrasound, and computed tomography [23,24,25]. However, some body structures, such as the hypopharynx, are technically difficult to illuminate accurately with such diffusers [5]. The LEL fiber, enabling uniform tumor illumination while minimizing exposure to non-tumor tissue, is expected to reduce the risk of edema and side effects on normal organs.

Limiting the area of illumination of NIR light may reduce the adverse effects of NIR-PIT. Although NIR-PIT is a highly tumor cell-selective treatment and has few adverse effects on normal cells, edema around the tumor has been reported after treatment [26,27,28]. When NIR light reacts with IR700 under oxygen-rich conditions, reactive oxygen species are generated, leading to edema in normal tissue. Moreover, since EGFR is expressed to some extent in normal mucosal cells, excess irradiation to normal areas might cause side effects if using an EGFR-targeting drug. The LEL fiber allows uniform light illumination of the entire tumor and reduces light exposure to non-tumor tissue. Therefore, it is expected that NIR-PIT with an LEL fiber is less likely to cause edema and side effects in normal organs.

Several limitations exist in this study. We used only one cell line. However, since the aim of this study was to evaluate the usefulness of a novel LEL fiber, it is important to determine if it can properly illuminate the NIR light. Our results clearly showed that an LEL fiber can be used for photoimmunotherapy. Moreover, since the cytotoxic mechanism of NIR-PIT involves membrane damage after NIR light exposure, the therapeutic effects of NIR-PIT are thought to occur independently of cell type if the NIR light is properly exposed. Second, various novel illumination devices for NIR-PIT, such as a dedicated catheter with light-emitting diodes, a temperature sensor, and an endovascular therapy-based light illumination technology, have been developed [29,30]. It is difficult to compare these reported methods with an LEL fiber. However, it is important to increase the number of illumination options to accommodate various tumors. We are convinced that the LEL fiber is at least suitable for irradiating small lumens and, therefore, there are many indications that exist.

## 5. Conclusions

Side-firing illumination with an LEL fiber may illuminate NIR light to only a certain area of a lateral wall in a narrow space. The use of NIR-PIT with an LEL fiber showed cytotoxic effects in vitro and suppressed tumor proliferation in vivo. These results indicated that an LEL fiber may be a new option for NIR light illumination in NIR-PIT.

## Data Availability

The data presented in this study are available on request from the corresponding author.

## References

[1] Mitsunaga M., Ogawa M., Kosaka N., Rosenblum L.T., Choyke P.L., Kobayashi H. (2011). Cancer cell-selective in vivo near infrared photoimmunotherapy targeting specific membrane molecules. Nat. Med..

[2] Kobayashi H., Choyke P.L. (2019). Near-Infrared Photoimmunotherapy of Cancer. Acc. Chem. Res..

[3] Gao X., Cui Y., Levenson R.M., Chung L.W., Nie S. (2004). In vivo cancer targeting and imaging with semiconductor quantum dots. Nat. Biotechnol..

[4] Tahara M., Okano S., Enokida T., Ueda Y., Fujisawa T., Shinozaki T., Tomioka T., Okano W., Biel M.A., Ishida K. (2021). A phase I, single-center, open-label study of RM-1929 photoimmunotherapy in Japanese patients with recurrent head and neck squamous cell carcinoma. Int. J. Clin. Oncol..

[5] Nishikawa D., Suzuki H., Beppu S., Terada H., Sawabe M., Hanai N. (2022). Near-Infrared Photoimmunotherapy for Oropharyngeal Cancer. Cancers.

[6] Nagaya T., Okuyama S., Ogata F., Maruoka Y., Choyke P.L., Kobayashi H. (2018). Endoscopic near infrared photoimmunotherapy using a fiber optic diffuser for peritoneal dissemination of gastric cancer. Cancer Sci..

[7] Okada R., Furusawa A., Inagaki F., Wakiyama H., Kato T., Okuyama S., Furumoto H., Fukushima H., Choyke P.L., Kobayashi H. (2021). Endoscopic near-infrared photoimmunotherapy in an orthotopic head and neck cancer model. Cancer Sci..

[8] Fukushima H., Turkbey B., Pinto P.A., Furusawa A., Choyke P.L., Kobayashi H. (2022). Near-Infrared Photoimmunotherapy (NIR-PIT) in Urologic Cancers. Cancers.

[9] Furumoto H., Kato T., Wakiyama H., Furusawa A., Choyke P.L., Kobayashi H. (2022). Endoscopic Applications of Near-Infrared Photoimmunotherapy (NIR-PIT) in Cancers of the Digestive and Respiratory Tracts. Biomedicines.

[10] Yano T., Minamide T., Takashima K., Nakajo K., Kadota T., Yoda Y. (2021). Clinical Practice of Photodynamic Therapy Using Talaporfin Sodium for Esophageal Cancer. J. Clin. Med..

[11] Overholt B.F., Lightdale C.J., Wang K.K., Canto M.I., Burdick S., Haggitt R.C., Bronner M.P., Taylor S.L., Grace M.G., Depot M. (2005). Photodynamic therapy with porfimer sodium for ablation of high-grade dysplasia in Barrett’s esophagus: International, partially blinded, randomized phase III trial. Gastrointest. Endosc..

[12] Zimmermann M., Zouhair A., Azria D., Ozsahin M. (2006). The epidermal growth factor receptor (EGFR) in head and neck cancer: Its role and treatment implications. Radiat. Oncol..

[13] Grandis J.R., Tweardy D.J. (1993). Elevated levels of transforming growth factor alpha and epidermal growth factor receptor messenger RNA are early markers of carcinogenesis in head and neck cancer. Cancer Res..

[14] Carpenter G., Cohen S. (1979). Epidermal growth factor. Annu. Rev. Biochem..

[15] Miyoshi Y., Nishimura T., Shimojo Y., Okayama K., Awazu K. (2023). Endoscopic image-guided laser treatment system based on fiber bundle laser steering. Sci. Rep..

[16] Kostic S.V., Rice T.W., Baker M.E., Decamp M.M., Murthy S.C., Rybicki L.A., Blackstone E.H., Richter J.E. (2000). Timed barium esophagogram: A simple physiologic assessment for achalasia. J. Thorac. Cardiovasc. Surg..

[17] Takashima K., Koga Y., Anzai T., Migita K., Yamaguchi T., Ishikawa A., Sakashita S., Yasunaga M., Yano T. (2022). Evaluation of Fluorescence Intensity and Antitumor Effect Using Real-Time Imaging in Photoimmunotherapy. Pharmaceuticals.

[18] Schindelin J., Arganda-Carreras I., Frise E., Kaynig V., Longair M., Pietzsch T., Preibisch S., Rueden C., Saalfeld S., Schmid B. (2012). Fiji: An open-source platform for biological-image analysis. Nat. Methods.

[19] Ogawa M., Takakura H. (2021). Photoimmunotherapy: A new cancer treatment using photochemical reactions. Bioorg. Med. Chem..

[20] Maruoka Y., Nagaya T., Nakamura Y., Sato K., Ogata F., Okuyama S., Choyke P.L., Kobayashi H. (2017). Evaluation of Early Therapeutic Effects after Near-Infrared Photoimmunotherapy (NIR-PIT) Using Luciferase-Luciferin Photon-Counting and Fluorescence Imaging. Mol. Pharm..

[21] Dumoulin F.L., Hildenbrand R., Oyama T., Steinbruck I. (2021). Current Trends in Endoscopic Diagnosis and Treatment of Early Esophageal Cancer. Cancers.

[22] Tang Y., Anandasabapathy S., Richards-Kortum R. (2021). Advances in optical gastrointestinal endoscopy: A technical review. Mol. Oncol..

[23] Omura G., Honma Y., Matsumoto Y., Shinozaki T., Itoyama M., Eguchi K., Sakai T., Yokoyama K., Watanabe T., Ohara A. (2023). Transnasal photoimmunotherapy with cetuximab sarotalocan sodium: Outcomes on the local recurrence of nasopharyngeal squamous cell carcinoma. Auris Nasus Larynx.

[24] Okamoto I., Okada T., Tokashiki K., Tsukahara K. (2022). A Case Treated With Photoimmunotherapy Under a Navigation System for Recurrent Lesions of the Lateral Pterygoid Muscle. In Vivo.

[25] Koyama S., Ehara H., Donishi R., Morisaki T., Ogura T., Taira K., Fukuhara T., Fujiwara K. (2023). Photoimmunotherapy with surgical navigation and computed tomography guidance for recurrent maxillary sinus carcinoma. Auris Nasus Larynx.

[26] Nakajima K., Sugikawa A., Yasui H., Higashikawa K., Suzuki C., Natsume T., Suzuki M., Takakura H., Tomita M., Takahashi S. (2023). In vivo imaging of acute physiological responses after treatment of cancer with near-infrared photoimmunotherapy. Mol. Imaging Biol..

[27] Cognetti D.M., Johnson J.M., Curry J.M., Kochuparambil S.T., McDonald D., Mott F., Fidler M.J., Stenson K., Vasan N.R., Razaq M.A. (2021). Phase 1/2a, open-label, multicenter study of RM-1929 photoimmunotherapy in patients with locoregional, recurrent head and neck squamous cell carcinoma. Head. Neck.

[28] Kato T., Okada R., Goto Y., Furusawa A., Inagaki F., Wakiyama H., Furumoto H., Daar D., Turkbey B., Choyke P.L. (2021). Electron Donors Rather Than Reactive Oxygen Species Needed for Therapeutic Photochemical Reaction of Near-Infrared Photoimmunotherapy. ACS Pharmacol. Transl. Sci..

[29] Hirata H., Kuwatani M., Nakajima K., Kodama Y., Yoshikawa Y., Ogawa M., Sakamoto N. (2021). Near-infrared photoimmunotherapy (NIR-PIT) on cholangiocarcinoma using a novel catheter device with light emitting diodes. Cancer Sci..

[30] Tsukamoto T., Fujita Y., Shimogami M., Kaneda K., Seto T., Mizukami K., Takei M., Isobe Y., Yasui H., Sato K. (2022). Inside-the-body light delivery system using endovascular therapy-based light illumination technology. eBioMedicine.

